# Identification of Cardiac Magnetic Resonance Imaging Thresholds for Risk Stratification in Pulmonary Arterial Hypertension

**DOI:** 10.1164/rccm.201909-1771OC

**Published:** 2020-02-15

**Authors:** Robert A. Lewis, Christopher S. Johns, Marcella Cogliano, David Capener, Euan Tubman, Charlie A. Elliot, Athanasios Charalampopoulos, Ian Sabroe, A. A. Roger Thompson, Catherine G. Billings, Neil Hamilton, Kathleen Baster, Peter J. Laud, Peter M. Hickey, Jennifer Middleton, Iain J. Armstrong, Judith A. Hurdman, Allan Lawrie, Alexander M. K. Rothman, Jim M. Wild, Robin Condliffe, Andrew J. Swift, David G. Kiely

**Affiliations:** ^1^ Sheffield Pulmonary Vascular Disease Unit, Sheffield Teaching Hospitals NHS Foundation Trust, Royal Hallamshire Hospital, Sheffield, United Kingdom; ^2^ Department of Infection, Immunity and Cardiovascular Disease, University of Sheffield Medical School, Sheffield, United Kingdom; and; ^3^ Statistical Services Unit, School of Mathematics and Statistics and; ^4^ Insigneo Institute for *In Silico* Medicine, University of Sheffield, Sheffield, United Kingdom

**Keywords:** risk stratification, pulmonary arterial hypertension, imaging, disease severity, prognosis

## Abstract

**Rationale:** Pulmonary arterial hypertension (PAH) is a life-shortening condition. The European Society of Cardiology and European Respiratory Society and the REVEAL (North American Registry to Evaluate Early and Long-Term PAH Disease Management) risk score calculator (REVEAL 2.0) identify thresholds to predict 1-year mortality.

**Objectives:** This study evaluates whether cardiac magnetic resonance imaging (MRI) thresholds can be identified and used to aid risk stratification and facilitate decision-making.

**Methods:** Consecutive patients with PAH (*n* = 438) undergoing cardiac MRI were identified from the ASPIRE (Assessing the Spectrum of Pulmonary Hypertension Identified at a Referral Center) MRI database. Thresholds were identified from a discovery cohort and evaluated in a test cohort.

**Measurements and Main Results:** A percentage-predicted right ventricular end-systolic volume index threshold of 227% or a left ventricular end-diastolic volume index of 58 ml/m^2^ identified patients at low (<5%) and high (>10%) risk of 1-year mortality. These metrics respectively identified 63% and 34% of patients as low risk. Right ventricular ejection fraction >54%, 37–54%, and <37% identified 21%, 43%, and 36% of patients at low, intermediate, and high risk, respectively, of 1-year mortality. At follow-up cardiac MRI, patients who improved to or were maintained in a low-risk group had a 1-year mortality <5%. Percentage-predicted right ventricular end-systolic volume index independently predicted outcome and, when used in conjunction with the REVEAL 2.0 risk score calculator or a modified French Pulmonary Hypertension Registry approach, improved risk stratification for 1-year mortality.

**Conclusions:** Cardiac MRI can be used to risk stratify patients with PAH using a threshold approach. Percentage-predicted right ventricular end-systolic volume index can identify a high percentage of patients at low-risk of 1-year mortality and, when used in conjunction with current risk stratification approaches, can improve risk stratification. This study supports further evaluation of cardiac MRI in risk stratification in PAH.

**At a Glance Commentary**

**Scientific Knowledge on the Subject**

Cardiac magnetic resonance imaging (MRI) is the gold standard test to assess right ventricular function and has a prognostic role in pulmonary arterial hypertension.

**What This Study Adds to the Field**

Improvement and maintenance of a low-risk profile (<5% mortality at 1 yr) is the current goal of treatment strategies in patients with pulmonary arterial hypertension. We identified and tested cardiac MRI thresholds to risk stratify patients at baseline and follow-up. Cardiac MRI can identify a high percentage of patients at low-risk of 1-year mortality when used as a sole risk-stratification tool and can improve risk stratification when used in conjunction with current risk-stratification approaches.

Pulmonary arterial hypertension (PAH) is a rare and life-shortening condition (1). Without treatment, life expectancy is less than 3 years (2), but with therapy, 5-year survival exceeds 60% in patients with idiopathic PAH (3–5). Current licensed therapies directed at the pulmonary vasculature target three pathways (6). There is evidence of superiority of upfront dual (7) or sequential oral combination therapy (8, 9) over oral monotherapy, but decisions regarding escalation to more intensive treatments, as well as transplantation, can be challenging.

The European Society of Cardiology and European Respiratory Society (ESC/ERS) guidelines propose a “traffic light” risk-stratification score to aid physicians in treatment decisions, categorizing patients as having low risk (“green,” <5%), intermediate risk (“amber,” 5–10%), or high risk (“red,” >10%) of mortality at 1 year based on a number of modifiable variables (10). These include an assessment of symptoms, exercise capacity, and right ventricular function (10, 11). Although the thresholds used for these variables were largely based on expert opinion, they have since been validated for PAH in three European registries (11–13). These studies demonstrated that patients who improved to a low-risk profile at follow-up had better outcomes than those who did not improve. Outcomes for patients remaining in the intermediate-risk group were significantly worse than those for low-risk patients, and current approaches aim to improve and maintain patients in the low-risk group (10, 14). Similar results were observed in the REVEAL (North American Registry to Evaluate Early and Long-Term PAH Disease Management) risk score (15). Risk-stratification approaches typically include multiple variables (10, 11); however, right ventricular function is thought to be the primary determinant of morbidity and mortality in PAH (16, 17). Cardiac magnetic resonance imaging (MRI) is the recognized gold standard for assessment of right ventricular function (18–20). Although echocardiography is more widely available, cheaper, and the most commonly used imaging modality for right ventricular assessment, it is limited by lack of inter- and intraobserver reproducibility (21) and acoustic windows (20, 22, 23).

Whereas studies have demonstrated the prognostic value of cardiac MRI in PAH (17, 24–27), cardiac changes evaluated by current risk-stratification tools are limited to atrial and pericardial assessment. Cardiac MRI metrics, which are known to be prognostic in PAH, are not included in either the REVEAL 2.0 or ESC/ERS risk scores (10, 28). We sought to identify whether cardiac MRI metrics could accurately risk stratify patients using the ESC/ERS criteria of three levels of risk and whether thresholds could be used to aid risk stratification. Some results of this study were reported previously in the form of an abstract (29).

## Methods

Patients with PAH were identified from the ASPIRE (Assessing the Spectrum of Pulmonary Hypertension Identified at a Referral Center) cardiac MRI database between April 2012 and March 2017. Patients underwent systematic evaluation (30, 31), including echocardiography, blood testing, exercise testing, lung function testing, multimodality imaging, and right heart catheterization. Patients were required to have mean pulmonary artery pressure ≥25 mm Hg and to have had pulmonary arterial wedge pressure recorded and were excluded if pulmonary arterial wedge pressure was >15 mm Hg. Patients with coexisting lung disease or left heart disease were excluded. Patients were listed in date order of cardiac MRI scan and assigned in alternate date order to the discovery or test cohort.

### MRI Acquisition and Analysis

Cardiac cine MRI was performed on a 1.5-T GE HDx MRI scanner using an eight-channel receiver array and multislice balanced steady-state imaging with retrospective gating (20 frames per cardiac cycle; slice thickness, 8 mm; field of view, 48 cm; matrix, 256 × 256; bandwidth, 125 kHz/pixel; repetition time/echo time, 3.7/1.6 ms). A stack of images in the short-axis plane with slice thickness of 8 mm (2-mm interslice distance) was acquired covering both ventricles from base to apex. End systole was considered to be the smallest cavity area. End diastole was defined as the first cine phase of the R-wave–triggered acquisition or largest volume.

Image analysis was performed by operators blinded to diagnosis and cardiac catheter result. Endocardial and epicardial surfaces were manually traced on short-axis imaging to obtain end-systolic and end-diastolic volumes for the right and left ventricles. Trabeculations for the chambers were not separately traced and were included as part of the volume cavity measurement (32). Right ventricular ejection fraction (RVEF) was calculated from these volume measurements as described previously (33). Volumes were indexed for body surface area and then corrected for age and sex and displayed as percentage predicted, as described previously (34). Ventricular mass index was calculated as right ventricular mass divided by left ventricular mass (35). Pulmonary artery relative area change was measured on the magnitude images of phase contrast images and calculated as the difference of maximum pulmonary arterial area minus minimal pulmonary arterial area, divided by minimal pulmonary arterial area. Phase contrast imaging parameters were as follows: repetition time/echo time, 5.6/2.7 ms; slice thickness, 10 mm; field of view, 48 cm, bandwidth, 62.5 kHz; matrix, 256 3128; 40 reconstructed cardiac phases; and velocity encoding of flow, 150 cm/s. Patients were in the supine position with a surface coil and with retrospective ECG gating.

### Statistical Analysis

Statistical analysis was performed using IBM SPSS version 25 and R software. In this article, a *quintile* refers to values that divide a ranked population into five equal groups. A *quintile group* refers to the population defined by the quintile. Data for continuous variables are presented as mean ± SD, and categorical data are presented as absolute values. Once thresholds were derived, volumes and values expressed as percentage predicted were rounded to the nearest whole integer. Patients who had undergone lung transplantation were censored at the time of surgery; all other surviving patients were censored on February 28, 2019. Survival curves were assessed using the log-rank test.

### Discovery Cohort

In the discovery cohort, cardiac MRI parameters were assessed for significance for 1-year mortality using univariate Cox regression analysis. A *P* value of <0.05 was considered statistically significant. Metrics significant at univariate analysis were used to identify thresholds. To identify thresholds, continuous variables were categorized into quintile groups for each metric. The quintiles were used as inclusive thresholds and were taken to two decimal places unless further precision was required. In each quintile group, 1-year percentage mortality was calculated. Contiguous quintile groups sharing the same level of 1-year mortality (<5%, 5–10%, and >10%) were combined.

### Test Cohort

The derived thresholds were applied to the test cohort. For each metric, if the corresponding group did not have the same level of risk, no further analysis was undertaken (ie, only those variables that showed consistency of risk were retained).

### Whole Cohort

Univariate and multivariate analyses of demographics, hemodynamics, and MRI parameters were performed using Cox regression analysis. Locally estimated scatterplot smoothing regression analysis was performed on the whole cohort for percentage mortality at 1 year for MRI metrics in which consistency of risk was identified.

### Follow-up Data

Follow-up data were obtained for patients who had undergone further cardiac MRI after a minimum of 3 months and before March 31, 2017.

### Combination with Other Risk-Stratification Tools

The REVEAL 2.0 risk score and number of low-risk criteria by the FPHR (French Pulmonary Hypertension Registry) approach were calculated to determine whether cardiac MRI could further stratify risk. This was performed in the incident population, maximizing available data for each risk score. For the FPHR approach, the distance threshold of >440 m for the 6-minute walking test was substituted with a distance threshold of >330 m for a low-risk incremental shuttle walking test (36, 37). Receiver operating characteristic curve analysis was performed to compare approaches to risk stratification.

### Mortality Data

Mortality data were obtained from the NHS Personal Demographics Service, for which electronic records are automatically updated when a death is registered in the United Kingdom. All patients on PAH therapies were followed up at our center as part of the national service specification for patients with pulmonary hypertension. No patients were lost to follow-up.

Ethics approval was granted by the local ethics committee (STH14169; “Assessing outcomes in patients with PAH using cardiac MRI,” ASPIRE Study 004).

## Results

**Figure 1.**
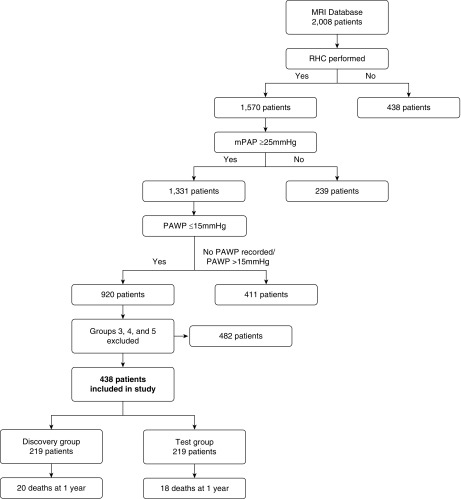
Flowchart showing patients included and reasons for exclusion. mPAP = mean pulmonary artery pressure; MRI = magnetic resonance imaging; PAWP = pulmonary arterial wedge pressure; RHC = right heart catheterization.

Between April 2012 and March 2017, 2,008 patients were identified and 438 had PAH and met inclusion criteria (Figure 1). Demographic data are displayed in Table 1. At the time of cardiac MRI, 51% of patients were incident and treatment naïve and 49% were prevalent. The mean age was 56.6 years, and 75% were female. The majority (85%) had idiopathic PAH, heritable PAH, or PAH in association with connective tissue disease. Portopulmonary hypertension and PAH in association with congenital heart disease composed 14% of patients. The remainder had PAH related to HIV infection or induced by drugs and toxins.

**Table 1.** Baseline Demographics

	All Patients (*n* = *438*)	Discovery (*n* = *219*)	Test (*n* = *219*)
Demographics			
Age, yr	56.6 ± 15.9	56.0 ± 15.7	57.2 ± 16.1
Sex, F/M (F %)	327/111 (75)	161/58 (74)	166/53 (76)
WHO FC I, *n* (%)	7 (2)	4 (2)	3 (1)
WHO FC II, *n* (%)	118 (27)	59 (27)	59 (27)
WHO FC III, *n* (%)	261 (60)	128 (58)	133 (61)
WHO FC IV, *n* (%)	52 (12)	28 (13)	24 (11)
Incident/prevalent (I%)	225/213 (51)	109/110 (50)	116/103 (53)
ISWD, m	231 ± 194	237 ± 196	223 ± 193
PAH subtype, *n* (%)			
IPAH/HPAH	197 (45)	101 (46)	96 (44)
PAH-CTD	163 (37)	78 (36)	85 (39)
PAH-CHD	38 (9)	18 (8)	20 (9)
PoPH	25 (6)	16 (7)	9 (4)
Other	15 (3)	6 (3)	9 (4)
Maximal treatment, *n* (%)			
None targeted	23 (5)	12 (6)	11 (5)
Oral monotherapy	100 (23)	46 (21)	54 (25)
Oral combination	205 (47)	101 (46)	104 (47)
Prostanoid with or without oral	110 (25)	60 (27)	50 (23)
Hemodynamics*			
mRAP, mm Hg	10 ± 5	10 ± 5	10 ± 5
mPAP, mm Hg	48 ± 14	47 ± 14	48 ± 15
PAWP, mm Hg	11 ± 3	11 ± 3	11 ± 3
Cardiac output, L/min	5.0 ± 1.8	5.1 ± 1.8	4.9 ± 1.8
Cardiac index, L/min/m^2^	2.8 ± 1.0	2.8 ± 1.0	2.7 ± 1.0
PVR, dyn · s · cm^−5^	711 ± 447	688 ± 432	735 ± 462
S$\bar{\text{v}}$_O_2__, %	65.4 ± 10.1	66.3 ± 9.9	64.5 ± 10.3
Survival analysis			
Deceased at 1 yr after cardiac MRI, *n* (%)	38 (8)	20 (9)	18 (8)

*Definition of abbreviations*: HPAH = heritable pulmonary arterial hypertension; IPAH = idiopathic pulmonary arterial hypertension; ISWD = incremental shuttle walking test distance; mPAP = mean pulmonary arterial pressure; mRAP = mean right atrial pressure; MRI = magnetic resonance imaging; PAH = pulmonary arterial hypertension; PAH-CHD = PAH associated with congenital heart disease; PAH-CTD = PAH associated with connective tissue disease; PAWP = pulmonary arterial wedge pressure; PoPH = portopulmonary hypertension; PVR = pulmonary vascular resistance; S$\bar{\text{v}}$_O_2__ = mixed venous oxygen saturation; WHO FC = World Health Organization functional class.

Data are shown as mean ± SD unless otherwise indicated. For all continuous variables, the difference between 95% confidence intervals for the groups included the value zero.

* Measured at right heart catheterization.

During the course of the study, 72% of patients received combination therapy (Table 1). A small number of patients, including nitric oxide vasoresponders maintained on calcium channel blockers, did not receive targeted treatment. Discovery and test cohorts were well matched, and confidence intervals (CIs) included the value zero for all continuous variables including cardiac MRI metrics (Tables 1 and 2).

**Table 2.** Cardiac Magnetic Resonance Imaging Metrics

Metric	All Patients (*n* = *438*)	Discovery (*n* = *219*)	Test (*n* = *219*)
Right-sided measurements			
RVESVi, ml/m^2^	53.0 ± 27.0	54.5 ± 26.9	51.5 ± 27.1
RVEDVi, ml/m^2^	88.6 ± 33.5	89.9 ± 33.7	87.3 ± 33.7
RVESVi %pred	220.0 ± 117.7	225.6 ± 122.7	214.4 ± 112.5
RVEDVi %pred	120.4 ± 45.3	121.9 ± 46.7	118.9 ± 44.0
RVEF, %	42.0 ± 13.3	41.0 ± 13.6	43.0 ± 13.0
RVEF %pred	62.7 ± 20.1	61.4 ± 20.7	64.0 ± 19.4
Left-sided measurements			
LVESVi, ml/m^2^	17.3 ± 8.2	17.9 ± 9.1	16.7 ± 7.1
LVEDVi, ml/m^2^	54.0 ± 16.1	54.6 ± 17.0	53.4 ± 15.1
LVESVi %pred	71.8 ± 33.8	74.1 ± 38.3	69.6 ± 28.4
LVEDVi %pred	73.0 ± 21.5	73.5 ± 22.8	72.5 ± 20.2
LVEF, %	68.3 ± 10.0	67.7 ± 10.6	68.9 ± 9.4
LVEF %pred	100.9 ± 14.7	100.2 ± 15.7	101.7 ± 13.7
Miscellaneous			
VMI	0.529 ± 0.297	0.528 ± 0.296	0.531 ± 0.299
PA relative area change, %	11.99 ± 8.70	12.39 ± 9.20	11.57 ± 8.15

*Definition of abbreviations*: %pred = percentage predicted for age and sex; LVEDVi = left ventricular end-diastolic volume index; LVEF = left ventricular ejection fraction; LVESVi = left ventricular end-systolic volume index; PA = pulmonary artery; RVEDVi = right ventricular end-diastolic volume index; RVEF = right ventricular ejection fraction; RVESVi = right ventricular end-systolic volume index; VMI = ventricular mass index.

Data are shown as mean ± SD. For all variables, the difference between 95% confidence intervals for the groups included the value zero.

### Survival Analysis

At 1 year after cardiac MRI, 38 patients (8.7%) had died; 20 patients (9.1%) were in the discovery cohort and 18 patients (8.2%) were in the test cohort. Patients older than 50 years were more likely to die at 1 year (*P* = 0.008), as were those with PAH associated with connective tissue disease (*P* = 0.001). Univariate and multivariate analyses for the whole cohort are shown in Table E1 in the online supplement. Results of Cox regression analysis for cardiac MRI variables in the discovery cohort, based on survival at 1 year, are shown in Table 3.

**Table 3.** Univariate Analysis in Discovery Cohort

Metric	Univariate Hazard Ratio	*P* Value	*n*
RVESVi, ml/m^2^	1.016	0.022	219
RVEDVi, ml/m^2^	1.009	0.150	219
RVESVi %pred	1.004	0.002	219
RVEDVi %pred	1.008	0.045	219
RVEF, %	0.955	0.008	219
RVEF %pred	0.966	0.003	219
LVESVi, ml/m^2^	0.935	0.055	219
LVEDVi, ml/m^2^	0.936	0.000	219
LVESVi %pred	0.987	0.113	219
LVEDVi %pred	0.957	0.000	219
LVEF, %	0.974	0.142	219
LVEF %pred	0.980	0.085	219
VMI	2.728	0.140	210
PA relative area change, %	0.956	0.121	219

For definition of abbreviations, *see* Table 2.

### Discovery and Test Cohorts

**Figure 2.**
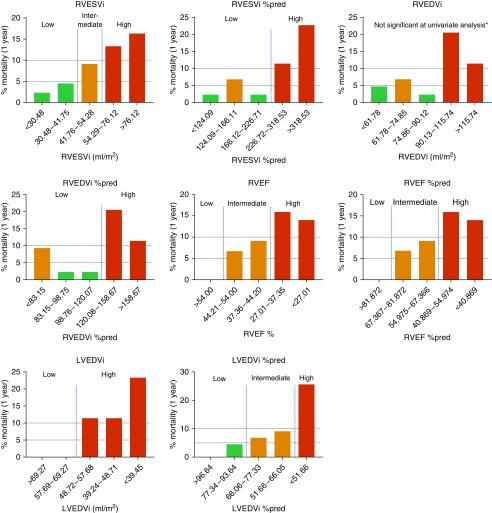
Histograms displaying quintile groups and percentage of mortality at 1 year for cardiac magnetic resonance imaging variables (discovery). Blue dashed lines represent division into categories of risk. *Division into risk categories was not performed. %pred = percentage predicted for age and sex; LVEDVi = left ventricular end-diastolic volume index; RVEDVi = right ventricular end-diastolic volume index; RVEF = right ventricular ejection fraction; RVESVi = right ventricular end-systolic volume index.

Quintile groups for each cardiac metric in the discovery group are displayed in Table E2. Calculated 1-year mortality per quintile group is presented in graphical form (Figure 2).

Derived thresholds were applied to the test cohort (Table E3). Levels of risk were nonconcordant between the discovery and test cohorts for right ventricular end-systolic volume index (RVESVi), percentage-predicted right ventricular end-diastolic volume index, percentage-predicted RVEF, and percentage-predicted left ventricular end-diastolic volume index (LVEDVi) and were excluded from further analysis.

**Figure 3.**
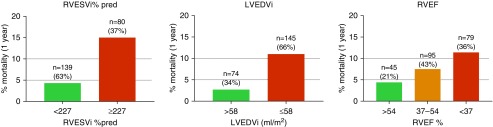
Histograms displaying percentage mortality at 1 year for derived thresholds for cardiac magnetic resonance imaging variables (test cohort). % pred = percentage predicted for age and sex; LVEDVi = left ventricular end-diastolic volume index; RVEF = right ventricular ejection fraction; RVESVi = right ventricular end-systolic volume index.

Three variables were concordant for levels of risk. Using a threshold of percentage-predicted RVESVi of 227%, patients could be stratified into a low-risk group (1-yr mortality, 4.3%), and a high-risk group (1-yr mortality, 15%). The low-risk group (those with percentage-predicted RVESVi below this threshold) represented 63% of the test cohort (56% of incident patients and 72% of prevalent patients) (Figure 3). Left ventricular end-diastolic volume index stratified patients into a low-risk group (1-yr mortality, 2.7%), and high-risk group (1-yr mortality, 11%) using a threshold of 58 ml/m^2^. The low-risk group (those with an LVEDVi above this threshold) represented 34% of the test cohort (26% of incident patients and 43% of prevalent patients). RVEF stratified patients into low (1-yr mortality, 4.4%)-, intermediate (7.4%)-, and high (11.4%)-risk groups using thresholds of >54%, 37–54%, and <37%, respectively. Using RVEF, 21% of the test cohort was identified as low risk, 43% as intermediate risk, and 36% as high risk.

When the analysis was repeated and patients with congenital heart disease were excluded, thresholds for RVEF and LVEDVi were unchanged. The threshold for percentage-predicted RVESVi changed from 227% to 225%.

There was no evidence of significant collinearity among LVEDVi, percentage-predicted RVESVi, and RVEF.

### Follow-up Data

**Figure 4.**
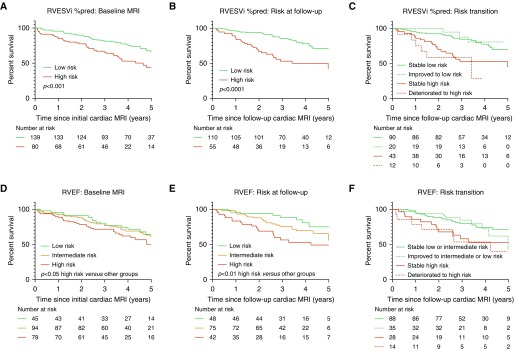
(*A–F*) Kaplan–Meier survival curves for RVESVi %pred at baseline (test cohort) (*A*), RVESVi %pred at follow-up cardiac magnetic resonance imaging (MRI) (*B*), transition of risk between baseline and follow-up cardiac MRI for RVESVi %pred (*C*), RVEF at baseline (test cohort) (*D*), RVEF at follow-up cardiac MRI (*E*), and transition of risk between baseline and follow-up cardiac MRI for RVEF (*F*). %pred = percentage predicted for age and sex; MRI = magnetic resonance imaging; RVEF = right ventricular ejection fraction; RVESVi = right ventricular end-systolic volume index.

Follow-up cardiac MRI was performed in 165 patients. At follow-up, percentage-predicted RVESVi and RVEF were able to risk stratify patients based on thresholds identified in the discovery cohort. Using percentage-predicted RVESVi for those who remained low risk (percentage-predicted RVESVi <227%) or improved to low risk (67% of patients in total), 1-year survival was 96.3%, whereas for those who remained high risk or deteriorated to high risk (33% of patients in total), 1-year survival was 87.3% (Figure 4). RVEF was able to stratify patients into high and low risk of 1-year mortality at follow-up. Patients with RVEF <37% (25% of patients) had 1-year survival of 83.3%, whereas patients with RVEF of 37% to 54% (45% of patients) and >54% (29% patients) had 1-year survival of 97.3% and 95.8%, respectively.

### Combining Cardiac MRI with REVEAL and FPHR Scores

**Figure 5.**
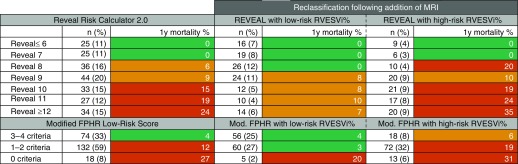
Impact of adding cardiac magnetic resonance imaging to REVEAL 2.0 and a modified FPHR (French Pulmonary Hypertension Registry) approach to European Society of Cardiology and European Respiratory Society guidelines for risk stratification. Green indicates low risk of 1-year mortality (<5%), amber indicates intermediate risk (5–10%), and red indicates high risk (>10%). Mod. = modified; MRI = magnetic resonance imaging; PAH = pulmonary arterial hypertension; REVEAL = North American Registry to Evaluate Early and Long-Term PAH Disease Management; RVESVi% = percentage-predicted right ventricular end-systolic volume index.

The REVEAL 2.0 and modified FPHR scores, in isolation and in combination with percentage-predicted RVESVi, are displayed for the incident population (*n* = 224) in Figure 5. For REVEAL 2.0, all patients had at least 11 of the 13 parameters available, and for modified FPHR, no data were missing. In those with a REVEAL 2.0 score ≥8 and in those determined to be either intermediate or high risk by the modified FPHR approach, percentage-predicted RVESVi reclassified patients into higher or lower risk categories (Figure 5).

Based on REVEAL 2.0 risk scores ≤6 for low risk, 7 to 8 for intermediate risk, and ≥9 for high risk (28) and using a dichotomized score of +2 or −2 for the percentage-predicted RVESVi threshold of 227%, MRI reclassified 47% of patients: 36% (*n* = 82) into a lower risk group and 11% (*n* = 25) into a higher risk group.

**Figure 6.**
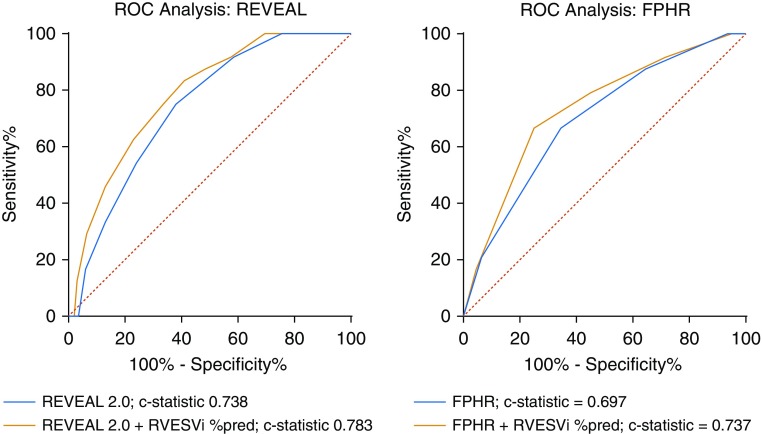
Receiver operating characteristic curves showing potential added value of cardiac magnetic resonance imaging in conjunction with the REVEAL 2.0 risk score calculator (left) and modified FPHR (French Pulmonary Hypertension Registry) approach to risk stratification (right), using an RVESVi percentage-predicted threshold of 227%. %pred = percentage predicted for age and sex; REVEAL = North American Registry to Evaluate Early and Long-Term PAH Disease Management; ROC = receiver operating characteristic; RVESVi = right ventricular end-systolic volume index.

Receiver operating characteristic curves are displayed in Figure 6. For the REVEAL 2.0 risk score, addition and subtraction of 2 points for a percentage-predicted RVESVi ≥227% and <227%, respectively, increased the C statistic from 0.74 (95% CI, 0.65–0.83) to 0.78 (95% CI, 0.70–0.87). Using the modified FPHR approach, the C statistic increased from 0.70 (95% CI, 0.59–0.80) to 0.74 (95% CI, 0.63–0.84) by adding an additional point for patients with a percentage-predicted RVESVi <227%.

### Verification of Thresholds

For LVEDVi, percentage-predicted RVESVi, and RVEF, locally estimated scatterplot smoothing regression analysis confirmed that identified thresholds were within the CIs for the associated risk of 1-year mortality (Figure E1).

## Discussion

To our knowledge, this study is the first to identify cardiac MRI thresholds that can be used to risk stratify patients with PAH according to the ESC/ERS “traffic light” approach at baseline and follow-up. Furthermore, the addition of percentage-predicted RVESVi independently predicted outcome and, when used in conjunction with current approaches, improved risk stratification.

A number of studies have demonstrated the prognostic value of cardiac MRI in PAH at baseline and follow-up (17, 24–27, 38). In addition to volumetric measurements, RVEF, right ventricular to pulmonary artery coupling metrics and pulmonary artery relative area change (17, 39–41) have also been shown to be prognostic. In this study, we confirmed the prognostic value of cardiac MRI, but, specifically, we identified and tested thresholds that may improve its clinical utility by allowing incorporation into risk stratification scores, which are increasingly used to inform treatment decisions (42).

RVEF is the most commonly reported cardiac MRI metric, and this study confirms its prognostic value but also identifies thresholds that can be used to identify patients at low risk (RVEF >54%), intermediate risk (RVEF 37–54%), and high risk (RVEF <37%) of 1-year mortality. The threshold of RVEF <35% in a previous study of 76 incident patients with PAH, identified as conferring a higher risk of mortality over a median follow-up period of 59 months (17), is similar to that identified in our study of <37% for high-risk patients. Using the ESC/ERS approach to risk stratification, 21%, 43%, and 36% of patients were identified at low, intermediate, and high risk of 1-year mortality, respectively, in the test cohort, similar to the results of approaches using multiple noninvasive and invasive parameters to assess risk (12, 13). Thresholds identified from this study for RVEF could be incorporated into the ESC/ERS risk stratification table to aid decision making.

In this study, baseline volumetric measurements of percentage-predicted RVESVi and LVEDVi were both prognostic. We previously demonstrated in a large study of 576 patients with PAH that percentage-predicted RVESVi was the cardiac MRI measurement with the strongest prognostic value (33), and in this study also independently predicted outcome. However, it is important to recognize that right ventricular volumes decrease with advancing age, are larger in men, and increase with body surface area (43). Our group and others have previously identified the need to adjust cardiac MRI variables for age, sex, and body surface area (33, 34, 44), and this study demonstrates for right ventricular volumes that correction for these variables is important when risk stratifying patients. In this study, pulmonary artery relative area change did not predict short-term mortality (1 yr), whereas it did predict mortality during the duration of this study (*P* < 0.005). This highlights that MRI metrics that measure right ventricular function may be more helpful in defining short-term mortality.

Although we were able to identify low, intermediate, and high risk of 1-year mortality for RVEF, we were able to identify only patients at low and high-risk of 1-year mortality using volumetric measurements of percentage-predicted RVESVi and LVEDVi. Thresholds for percentage-predicted RVESVi <227% and LVEDVi >58 ml/m^2^ identified low-risk patients. An LVEDVi threshold <40 ml/m^2^ was previously used to identify patients at increased risk of mortality (26), but our study suggests that an LV volume of ≤58 ml/m^2^ confers a high risk of mortality at 1 year. Using a threshold for percentage-predicted RVESVi <227% and for left ventricular volume of >58 ml/m^2^, 63% and 34% of patients, respectively, could be identified at low risk of 1-year mortality. RVEF identified 21% and 43% of patients as low and intermediate risk, respectively.

Treatment strategies are currently based on maintaining or improving patients to a low-risk group, and a dichotomized score identifying low- and high-risk groups may be a preferable approach to informing and simplifying treatment decisions. In particular, data from a number of studies have demonstrated that patients identified as intermediate risk using current risk stratification scores have significantly worse survival than low-risk patients. Identification and maintenance of a low-risk status remains the goal of current treatment strategies. A number of registry studies have shown that in patients whose risk profile can be modified to low risk, outcomes are significantly improved (13, 15). However, registry analysis using current approaches to risk stratification has identified only small numbers of patients in this low-risk category at presentation (12% of patients in COMPERA [Comparative, Prospective Registry of Newly Initiated Therapies for Pulmonary Hypertension] [13] and 23% in the Swedish registry); at follow-up, more patients achieved low-risk status (24% in COMPERA, 39% in the Swedish registry [12], and 42% in the French registry [11]). In contrast, in this study using percentage-predicted RVESVi, 63% of patients overall could be identified as low risk in the test cohort, with 56% of incident patients (newly diagnosed) and 72% of prevalent patients (following initiation of treatment). One-year mortality of all incident patients in this registry was 10.7% compared with 11.0% in COMPERA and 14% in the Swedish registry; for prevalent patients, 1-year mortality was 6.6% in our cohort, 10.3% in COMPERA, and 9% in the Swedish registry. Whereas the French registry included idiopathic, heritable and drug-induced PAH, the COMPERA, Swedish registry, and our own registry also included patients with other forms of PAH, predominantly related to connective tissue diseases. The hemodynamic parameters of our patients were similar to those in COMPERA and the French registry, although COMPERA includes a smaller proportion of patients in World Health Organization Functional Class II. The enrollment period in our study is more recent, and >70% of patients received combination therapy. Nonetheless, at diagnosis, cardiac MRI was able to identify a high percentage of patients at low risk (56%). Our findings suggest that assessing right ventricular function using gold standard techniques may be preferable to other investigative approaches if identification of patients at low risk of 1-year mortality is the goal. Further study is required to test this hypothesis. Using thresholds identified in the discovery cohort, we also demonstrated that cardiac MRI measurements can be used at follow-up to risk stratify patients and that improvement to low risk or maintenance of patients in a low-risk group is strongly prognostic.

Finally, we assessed how cardiac MRI could be used in conjunction with current risk-stratification approaches; percentage-predicted RVESVi, when used in conjunction with REVEAL 2.0 and modified FPHR, was able to further risk stratify patients. For patients classified as low risk by the modified FPHR or with a REVEAL 2.0 risk score ≤6, MRI did not affect risk stratification. However, when used in conjunction with the REVEAL 2.0 risk calculator and using a dichotomized score of +2 or −2 points for a percentage-predicted RVESVi threshold of 227%, for patients with a REVEAL 2.0 score >6, MRI was able to reclassify 36% of patients into a lower risk group and 11% of patients into a higher risk group.

### Limitations

Our approach to identifying thresholds for risk stratification using quintile groups in the discovery cohort is a valid but empiric approach. If the sample size were larger, other cardiac MRI parameters might have been identified. Like the COMPERA and Swedish registries, we included patients with congenital heart disease. Although repeated analysis with these patients excluded identified unchanged or similar thresholds, we recommend further study in congenital heart disease, and thresholds in this study should be used with caution in this population. The aim of this study was to assess whether a threshold approach could be used to risk stratify patients using MRI, and the methodology used may not have identified optimal thresholds. Our findings would benefit from external validation.

### Conclusions

Thresholds derived from cardiac MRI metrics may be used to risk stratify patients with PAH. This registry demonstrates that cardiac MRI, when used as a sole risk-stratification tool, identifies a high percentage of patients at low risk of 1-year mortality and can improve risk stratification when used in conjunction with current risk stratification approaches.

## Supplementary Material

Supplements
