# Supplementary material for: Identification of Cardiac Magnetic Resonance Imaging Thresholds for Risk Stratification in Pulmonary Arterial Hypertension
Source: Am J Respir Crit Care Med. 2020 Feb 15;201(4):458–68. doi: 10.1164/rccm.201909-1771OC (PMC7049935; doi:10.1164/rccm.201909-1771OC)
Supplement: Supplements [file rccm.201909-1771OC.html]

Identification of Cardiac Magnetic Resonance Imaging Thresholds for Risk Stratification in Pulmonary Arterial Hypertension | American Journal of Respiratory and Critical Care Medicine

- disclosures.pdf (475 KB)
- lewis\_data\_supplement.pdf (787 KB)
