# Supplementary material for: Identification of Cardiac Magnetic Resonance Imaging Thresholds for Risk Stratification in Pulmonary Arterial Hypertension
Source: Am J Respir Crit Care Med. 2020 Feb 15;201(4):458–68. doi: 10.1164/rccm.201909-1771OC (PMC7049935; doi:10.1164/rccm.201909-1771OC)
Supplement: Supplements [file rccm.201909-1771OC_lewis_data_supplement.pdf]

Identification of Cardiac MRI Thresholds for Risk Stratification in Pulmonary Arterial Hypertension

Robert A. Lewis MBChB, Christopher S. Johns PhD, Marcella Cogliano MSc,
David Capener MSc, Euan Tubman MBChB, Charlie A. Elliot MD,
Athanasios Charalampopoulos MD, Ian Sabroe PhD, A.A. Roger Thompson PhD,
Catherine G. Billings PhD, Neil Hamilton DPharm, Kathleen Baster MSc, Peter J. Laud MSc,
Peter M. Hickey MBChB, Jennifer Middleton MBChB, Iain J. Armstrong PhD,
Judith A. Hurdman MD, Allan Lawrie PhD, Alexander M.K. Rothman BMBCh, PhD,
Jim M. Wild PhD, Robin Condliffe MD, Andrew J. Swift PhD, and David G. Kiely MD

ONLINE DATA SUPPLEMENT

Online Data Supplement

Figure E1 (online supplement): LOESS regression curve fitted to the data demonstrating predicted mortality at one-year for a) LVEDVi, b) RVESVi %predicted, c) RVEF

LOESS = locally weighted scatterplot smoothing; LVEDVi = left ventricular end-diastolic volume, indexed for body surface area; RVESVi = right ventricular end-systolic volume indexed for body surface area; RVEF = right ventricular ejection fraction; %pred = displayed as percent predicted corrected for age and sex.

Table E1 (online supplement): Univariate and multivariate analysis on whole cohort (scaled)					
	n	Univariate Hazard Ratio	*P* value	Multivariate Hazard Ratio	*P* value
**Demographics**					
Age >50	438	3.576	0.008		
Sex (F)	438	0.562	0.086		
WHO FC I and II vs III and IV	430	2.724	0.037		
IPAH		0.534	0.079		
PAH-CTD		3.017	0.001		
PAH-CHD		0.195	0.269		
ISWD (m)	398	0.174	<0.001	0.162	<0.001
**Haemodynamics**					
mRAP (mmHg)	224	1.55	0.006		
mPAP (mmHg)	224	1.254	0.263		
SvO2 (%)	224	0.458	<0.001		
Cardiac Index (l/min/m^2)	224	0.506	0.016		
PVR (dynes/sec/cm^{-5})	224	1.5	0.06		
**Other parameters**					
eGFR <60 (ml/min/1.73m^2)	436	3.104	<0.001		
SBP <110 (mmHg)	217	1.025	0.968		
Heart Rate >96	417	4.123	0.001		
Pericardial effusion present	438	3.323	0.001		
Recent hospitalisation (6m)	438	1.822	0.071		
**MRI metrics**					
RVEDVi	438	1.285	0.07		
RVEDVI %pred	438	1.426	0.011		
RVESVi	438	1.392	0.011		
RVESVI %pred	438	1.616	<0.001	1.571	0.025
LVEDVi	438	0.451	<0.001		
LVEDVI %pred	438	0.435	<0.001		
LVESVi	438	0.601	0.016		
LVESVI %pred	438	0.598	0.066		
RVEF	438	0.634	0.005		
LVEF	438	0.847	0.275		
RVEF %pred	438	0.586	0.001		
LVEF %pred	438	0.795	0.16		
PA relative area change (%)	438	0.642	0.029		
VMI	438	1.125	0.446		

Hazard ratios for continuous variables are scaled by dividing individual values by the standard deviation (z-score). Due to the limited number of events, only 6 variables were entered into the multivariate analysis reflecting measures of symptoms, exercise capacity, markers of haemodynamic

severity and right ventricular function. WHO FC, ISWD, mRAP, Cardiac Index, RVESVi %pred and RVEF were significant at univariate analysis and were entered into the multivariate model.

WHO FC = World Health Organisation functional class; IPAH = idiopathic pulmonary arterial hypertension; PAH-CTD = pulmonary arterial hypertension associated with connective tissue disease; PAH-CHD = pulmonary arterial hypertension associated with congenital heart disease; ISWD = incremental shuttle walking test distance; mRAP = mean right atrial pressure; mPAP = mean pulmonary arterial pressure; SvO2 = mixed venous oxygen saturation; PVR = pulmonary vascular resistance; eGFR = estimated glomerular filtration rate; SBP = systolic blood pressure; RVEDVi = right ventricular end-diastolic volume, indexed for body surface area; RVESVi = right ventricular end-systolic volume, indexed for body surface area; LVEDVi = left ventricular end-diastolic volume, indexed for body surface area; LVESVi = left ventricular end-systolic volume, indexed for body surface area; LVEF = left ventricular ejection fraction; RVEF = right ventricular ejection fraction; PA = pulmonary artery; VMI = ventricular mass index; %pred = displayed as percent predicted for age and sex.

Table E2 (online supplement): Percentage mortality at 4, 6 and 12 months by quintile group				
	Discovery Cohort			
	n =	% mortality		
		4 months	6 months	12 months
**RVESVi (ml/m²)**				
**<30.48**	43	0	2.3	2.3
**30.48-41.75**	44	2.3	2.3	4.5
**41.76-54.28**	44	4.5	9.1	9.1
**54.29-76.12**	45	2.2	4.4	13.3
**>76.12**	43	7	14	16.3
**RVESVi %pred**				
**<124.09**	43	2.3	2.3	2.3
**124.09-166.11**	44	2.3	4.5	6.8
**166.12-226.71**	44	0	2.3	2.3
**226.72-318.53**	44	2.3	6.8	11.4
**>318.53**	44	9.1	15.9	22.7
**RVEDVi %pred**				
**<83.15**	43	4.7	9.3	9.3
**83.15-98.75**	44	0	0	2.3
**98.76-120.07**	44	0	2.3	2.3
**120.08-158.67**	44	9.1	13.6	20.5
**>158.67**	44	2.3	6.8	11.4
**RVEF %**				
**<27.01**	43	4.7	11.6	14
**27.01-37.35**	44	6.8	11.4	15.9
**37.36-44.20**	44	2.3	6.8	9.1
**44.21-54.00**	45	2.2	2.2	6.7
**>54.00**	43	0	0	0
**RVEF %pred**				
**<40.869**	43	4.7	11.6	14
**40.869-54.974**	42	4.5	11.4	15.9
**54.975-67.366**	44	4.5	6	9.1
**67.367-81.872**	43	2.3	2.3	6.8
**>81.872**	44	0	0	0
**LVEDVi (ml/m²)**				
**<39.24**	43	4.7	14	23.3
**39.24-48.71**	44	6.6	11.4	11.4
**48.72-57.68**	44	4.5	6.8	11.4
**57.69-69.27**	44	0	0	0
**>69.27**	44	0	0	0
**LVEDVi %pred**				
**<51.66**	43	7	16.3	25.6
**51.66-66.05**	44	6.8	9.1	9.1
**66.06-77.33**	44	2.3	4.5	6.8
**77.34-93.64**	44	0	2.3	4.5
**>96.64**	44	0	0	0

RVESVi = right ventricular end-systolic volume, indexed for body surface area; RVEDVi = right ventricular end-diastolic volume, indexed for body surface area; RVEF = right ventricular ejection fraction; LVEDVi = left ventricular end-diastolic volume, indexed for body surface area; %pred = displayed as percent predicted for age and sex.

Table E3 (online supplement): Percentage mortality at 4, 6 and 12 months					
	Test Cohort				Corresponding risk category in Discovery cohort
	n =	% mortality			
		4 months	6 months	12 months	
**RVESVi (ml/m²)**					
**<41.76**	100	2	4	6	Low (<5%)
**41.76-54.28**	35	0	0	0	Intermediate (5-
**>54.29**	84	8.3	8.3	14.3	High
**RVESVi %pred**					
**<226.72**	139	1.4	2.9	4.3	Low
**>226.72**	80	8.8	8.8	15	High
**RVEDVi %pred**					
**<120.08**	134	3	4.5	6	Low (<5%)
**>120.08**	85	5.9	5.9	11.8	High
**RVEF %**					
**<37.36**	79	6.3	6.3	11.4	High
**37.36-54.00**	95	3.2	5.3	7.4	Intermediate
**>54.00**	45	2.2	2.2	4.4	Low
**RVEF %pred**					
**<54.975**	76	7.9	7.9	13.2	High
**54.975-81.872**	101	1	3	5	Intermediate
**>81.872**	42	4.8	4.8	7.1	Low (<5%)
**LVEDVi (ml/m²)**					
**<57.69**	145	5.5	6.9	11	High
**>57.69**	74	1.4	1.4	2.7	Low
**LVEDVi %pred**					
**<51.66**	28	7.1	7.1	17.9	High
**51.66-77.33**	105	3.8	4.8	6.7	Intermediate
**>77.33**	86	3.5	4.7	7	Low (<5%)

RVESVi = right ventricular end-systolic volume, indexed for body surface area; RVEDVi = right ventricular end-diastolic volume, indexed for body surface area; RVEF = right ventricular ejection fraction; LVEDVi = left ventricular end-diastolic volume, indexed for body surface area; %pred = displayed as percent predicted for age and sex. Blue highlighting indicates non-concordance for levels of risk between discovery and test cohorts.

a)

b)

c)

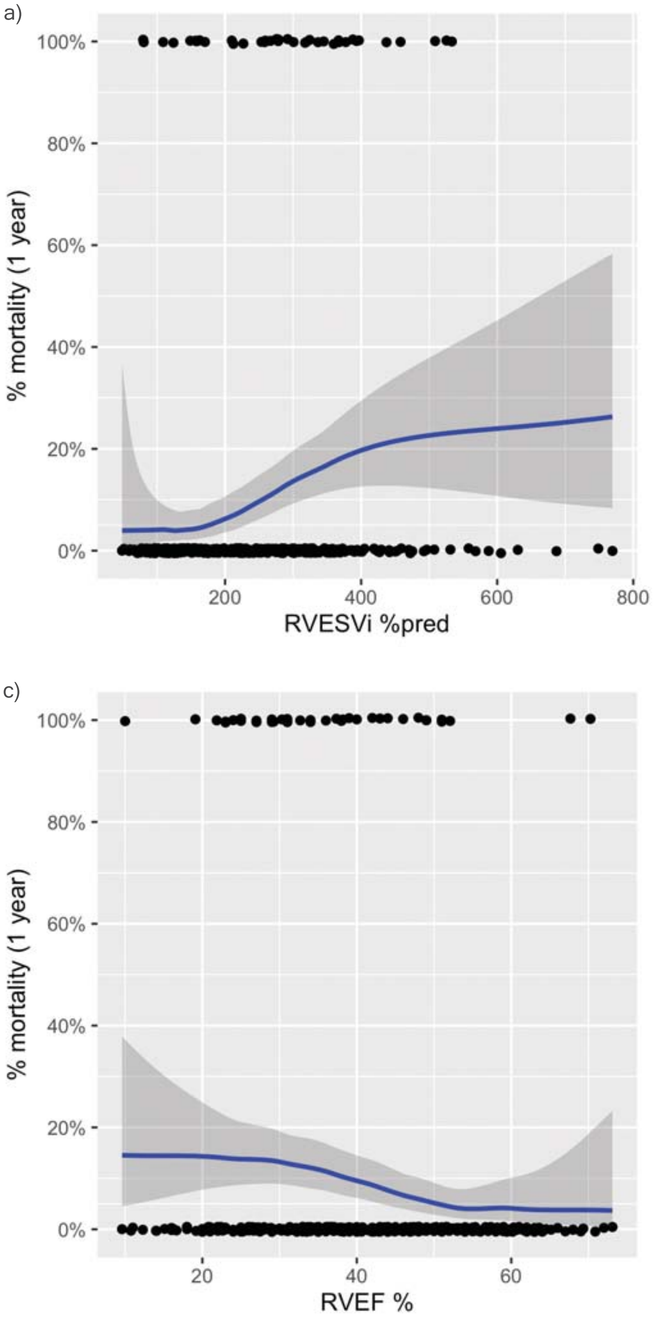
100%
80%
60%
40%
20%
0%
% mortality (1 year)
200
400
600
800
RVESVi %pred
30
60
90
LVEDVi (ml/m²)
20
40
60
RVEF %
